# The Parliamentary Inquiry Concerning Lunatics

**Published:** 1860-10-01

**Authors:** 


// THE JOURNAL
OF
PSYCHOLOGICAL MEDICINE
AND
MENTAL PATHOLOGY.

OCTOBER 1, 1860.

### Art. I.—THE PARLIAMENTARY INQUIRY CONCERNING LUNATICS.

The Parliamentary inquiry touching the care and treatment of lunatics, after having been protracted over three sessions, has at length terminated. The Select Committee have reported, and we proceed at once to an examination of that Report, simply premising that, in the session which has just terminated, additional evidence was given by Mr. Bolden, Dr. Hood, Mr. Perceval, and the Earl of Shaftesbury. Mr. Joseph Elmer, attached to the office of the Masters in Lunacy, was also examined. The last-named gentleman gave evidence chiefly on the expenses connected with lunacy inquisitions: Mr. Bolden detailed the particulars of a case of unjust confinement in a private lunatic asylum. He also laid before the Committee certain estimates of the probable expenses of establishments for private patients in this country, framed upon the plan of the chartered asylums of Scotland. Dr. Hood offered sundry suggestions for the more effective carrying out of the inspection and care of lunatics; Mr. Perceval corrected and explained one or two points in the evidence he had previously tendered; and the Earl of Shaftesbury stated the advantages attached to the present constitution and method of working of the Lunacy Commission, pointed out the serious disadvantages which he conceived would arise if any radical changes were made in the formation of the Commission, and indicated a mode in which its executive capacity might be largely increased.

The Report commences by stating that the number of lunatics, using the word in its statutory sense (that is to say, "every person being an idiot or lunatic, or of unsound mind, though that meaning will include many persons to whom the word is not strictly applicable"), "is very large, and it is to be feared that the number is still on the increase." Whether this number is increasing in a greater ratio than the increase of population, may, it is remarked, be doubtful; "as it should not be forgotten that old chronic cases, which were not formerly under supervision, have now, with the increase and improvement of public asylums, been since brought into them; and, in addition to this, the care of the patient is so much more efficient than it was before, that the annual mortality is considerably diminished, and the consequent longevity is considerably increased." (p. iii.) The Report then proceeds:—

"Until the year 1844, there were no data upon which an accurate opinion could be formed with reference to this important part of the subject; but since that year cases have been better looked up, and more closely attended to. Taking this as our starting-point, and comparing the number of patients on the 1st of January, 1844, with the number of patients on the 1st of January, 1858 and 1859, we find the following results:—

| | 1844. | 1858. | 1839. |
|---|---|---|---|
| Private patients in asylums, hospitals, and licensed houses . . . . . . . . . | 3,790 | 4,612 | 4,762 |
| Pauper lunatics and idiots in asylums, hospitals, and licensed houses . . . | 7,482 | 17,572 | 18,022 |
| Pauper lunatics, and idiots in workhouses and with friends, &c. . . . . . . . | 9,339 | 13,163 | 13,208 |
| Total . . . . . | 20,611 | 35,347 | 35,982 |

There was, therefore, on the 1st of January, 1858 and 1859, as compared with the 1st of January, 1844, an increase of about 15,000, out of totals of 35,347 and 35,982; an increase apparently very great in proportion to the increase which has taken place in the population during the same period. But it should be borne in mind that in the return for 1844 many patients living with their friends as paupers were not included, there being no record of them at that period. After making allowance for the operation of the causes above referred to, it is to be feared that a large part of the increase must still be attributed to other causes. Taking the figures as they stand, it is a melancholy fact that out of every 600 people in England and Wales, one at least is in such a state that, in many respects, he is incapable of managing himself and his affairs. A vast proportion, no doubt, are cases either of natural idiotcy or of mental imbecility arising from age, epilepsy, fits, and other causes, where the maladies may be regarded as chronic or incurable. With regard to them, little more can be done by any laws however wise, or any regulations however prudent, than to provide the patients with such comforts as their circumstances will admit; but with regard to others, since 50 or 60, or even 70 per cent. are capable of cure, if taken in time and carefully treated, it is certainly a matter of primary importance that our legislative provisions should be so framed as to promote the accomplishment of this desirable object." (pp. iii. iv.)

The provisions of the Acts of Parliament now in force which relate to lunatics in *public asylums* are next briefly recited, and the Report then states:—

"It appears from the evidence that these asylums are, generally speaking, so well looked after, and so carefully attended to, that, as regards them, but little alteration is required in the law. In some cases it may be a question whether they are not, in their structure, inconveniently large; whether the staff of attendants should not be increased; whether higher remuneration in some instances should not be given; and whether it might not be advisable to erect, in connexion with them, detached buildings, of a simple and inexpensive character, for the reception of imbecile and chronic patients. But these and the like matters require no alteration in the law, and may well be left to the visiting justices to regulate and determine, acting in communication with the Commissioners in Lunacy and the Secretary of State." (p. iv.)

The state of *lunatics confined in workhouses* is next considered. The number of these lunatics amounted, on the 1st of January, 1857, to 6800; and on the 1st of January, 1859, to 7632. The law relating to this class of lunatics, the Report states, "is certainly in a very unsatisfactory state." The Report then proceeds:—

"By the Poor Law Amendment Act the detention in any workhouse of 'any dangerous lunatic, insane person, or idiot,' for a longer period than fourteen days, is expressly prohibited; and the word 'dangerous' is read as applicable to each of the three classes of mentally disordered persons who are there mentioned. But with regard to those who are not dangerous, the statutory provisions are ambiguous. On the one hand, it seems to have been contemplated by the Legislature that all pauper lunatics should be sent to some asylum, registered hospital, or licensed house, under an order by a justice or justices; on the other hand, there are provisions in the same Act, and also in another Act of Parliament, passed in the same session, which seem to recognise, to a certain extent, the detention in workhouses of paupers deemed by law to be insane. The consequence is, that large numbers of pauper lunatics are kept in these houses without a certificate of their mental condition, and without an order from any magistrate regarding them as lunatics, although a large portion of such persons, especially in the rural districts, may be correctly described as harmless lunatics, who, if kept under a slight degree of supervision, are capable of useful and regular occupation, or whose infirmity of mind is consequent on epilepsy, or paralysis, or fatuity from old age. It cannot be denied that, with regard to those who are really lunatics, there is a great absence of proper supervision, attendance, and medical treatment. In some workhouses there are not even separate wards; mechanical restraint is frequently applied, because the imperfect state of the accommodation will not admit of a better mode of treatment; in many cases the medical officers of a union cannot have the special knowledge requisite for the management of the insane; and it may generally be concluded, that the special appliances of a union workhouse are not by any means equivalent as to this class of inmates to those of a lunatic asylum.

"The state of the law on this branch of the subject appears to require amendment. Your Committee are not prepared to recommend that all these cases, without exception, should be removed to asylums; but they are of opinion that no person should be detained in a workhouse respecting whose sanity a doubt exists, without a medical certificate, renewable quarterly, stating that the patient is a proper patient to be kept in the workhouse; that there should, if possible, be distinct wards for such patients, with distinct attendance; that the guardians of the union should specially visit such patients once in each quarter, and make a special entry on each such visit of their state and condition; that the Commissioners should also visit them at least once in each year, and that the same power of removing any patient to an asylum should be given to the Commissioners as that which the justices now have." (p. v.)

The clauses of the Lunatic Asylums' Bill brought before the House of Commons by Mr. Walpole, and referred to the Select Committee on Lunatics in the first Session of 1859 are next considered, although the Bill has not been re-introduced. The object of the Bill was—

"To facilitate the union of certain boroughs to counties, where the lunatics in such boroughs are now sent, for want of an asylum, to great distances from their own immediate neighbourhood; to enable the Secretary of State, where two or more counties have agreed to unite, but cannot agree about the plans and estimates of the intended asylum, to determine for them what plans and estimates shall be proceeded with and carried into execution; to authorize the committee of visitors to hire or take on lease land or buildings, either for the employment or occupation of the patients in the asylum, or for the temporary accommodation of any pauper lunatics for whom the accommodation in the asylum is inadequate; to authorize the committee of visitors to pay or contribute such sum of money as the Commissioners in Lunacy shall approve, for or towards the enlargement of any churchyard or consecrated public burial-ground, that the lunatics dying in any asylum need not be buried within the precincts of that asylum, to which in many cases great objections are entertained; to alter the interpretation of the word county, so as not to include under that term the county of a city or the county of a town; and to provide that a pauper lunatic found in any borough which is exempt from contributing to the county asylum shall be chargeable to the borough, and not to the county, when the settlement of such pauper lunatic cannot be ascertained." (pp. v. vi.)

The Committee concur in the general expediency of these alterations, and add :—

"It would further seem desirable to reduce the time at which committees of visitors may grant superannuation allowances to their medical officers. Their duties are so peculiar, and such painful consequences are known to result from incessant intercourse with the various forms of this distressing disease, when prolonged for many years, that your Committee believe it would tend to greater efficiency of service, if the period which stands at present at 20 years, were reduced to 15. It would also be desirable that the name of some relation of the patient should be inserted in the order of admission of a pauper lunatic into an asylum, to whom, in the case of the death of such patient while in the asylum, notice should be sent." (p. vi.)

The Acts of Parliament relating to lunatics in *private asylums* are now considered and their provisions briefly recapitulated. The Report then notices an opinion which had been submitted to the Committee that nothing short of the entire abolition of private asylums would be effectual in remedying their defects. The Report next refers to the recommendation of other witnesses, and particularly of Lord Shaftesbury, that "the magistrates should be empowered, if they think fit, to provide asylums by money raised on the security of the rates, for all classes of lunatics :"—

"The main reason for this suggestion is thus put by Lord Shaftesbury :—'When I look into the whole matter,' he says, 'I see that the principle of profit vitiates the whole thing; it is at the bottom of all those movements that we are obliged to counteract by complicated legislation, and if we could but remove that principle of making a profit, we should confer an inestimable blessing upon the middle classes, getting rid of half the legislation, and securing an admirable, sound, and efficient system of treatment of lunacy.' Again, in answer to a question whether he would have those asylums in every part of the kingdom, as there are public asylums for paupers, he adds, 'Yes; these asylums would be quite free from all those vicious motives that have been referred to in the licensed houses.' The examples which he would principally take as his guide are the chartered asylums dependent on charitable endowment, or private benefactions, in Scotland, of which there are seven, and the hospitals in England founded out of private funds, of which there are eleven." (p. vii.)

Upon this question the Committee conclude—

"The establishment of asylums of this character deserves to be encouraged, if it could be effected by private contributions. But should a power be given to establish such asylums throughout the kingdom as public institutions, by money to be raised on the security of a rate, the apprehension of a burden to be imposed on the ratepayers would, in the opinion of your Committee, render such an enactment inoperative; and they cannot recommend the establishment of them upon a compulsory system." (p. vii.)

The Committee assumed that "it would not be possible to abolish altogether the private asylums, or licensed houses," and it therefore became "all the more important to consider in what manner they can best be regulated." The suggestions submitted to the Committee are considered under four different heads.

1st. *The Suitableness of the House for the Purpose for which it is licensed.*—The Report states that it is to be feared that several of the houses, both in the metropolis and the country, are not well suited for the purpose; but "many which are unsuitable have had licences for years past, which have given to the property an additional value, and, therefore, it is generally difficult to refuse a renewal of them." And this does not constitute the chief obstacle in the way of improvement. "The great and leading difficulty," the Report continues, "is to find proper persons to undertake the charge of such an establishment. The fittest men may not have the capital, or those who have the capital are not the fittest men. The consequence is, that licences are given or continued to some capitalist, upon the condition that he has under him a medical superintendent; but as the superintendent has not the same control as the proprietor of the house, there is a diminished, or at any rate a divided responsibility, which cannot be otherwise than prejudicial to good management."

These defects are perfectly well known to the Commissioners and the magistrates, and much care is being devoted to their removal; but the Committee think that some amendment of the law might be advantageously introduced. It is therefore suggested that,—

"Except in cases to be specially allowed by the visitors or Commissioners, the proprietor, or, in the case of a joint ownership, one of the proprietors, should, as regards future licences, be required by law to reside on the spot. Nothing can lead to greater abuses than that large proprietors should have three or four houses, and reside in none of them. As a general rule, the proprietor, or one of the proprietors, ought to reside; and where he is permitted to be non-resident, the appointment of the medical superintendent should be subject to the approval of the Commissioners or visitors, as the case may be." (p. viii.)

2nd. *The Circumstances under which the Patient may be placed under Restraint, and the Safeguards provided for the Propriety of his Confinement.*—This portion of the Report is too important to be curtailed.

"This is by far the most difficult part of the subject. It has been suggested that in all cases the alleged lunatic, before he is confined, should, as a matter of right, be entitled to have his case tried and decided by some magistrate; or, as it has been proposed in a more mitigated form, that the medical certificates of the alleged insanity should be inspected and verified before a magistrate; and that if the magistrate was not satisfied with them, he should have the power of inquiring into the truth of the statement made, and of the necessity of the intended confinement. The exact nature of the former proposition, and the principal reasons upon which it is founded, are explained at length in the Second Report, in answer to question 179. The latter proposition would tend to assimilate the law of England to the law at present existing in Scotland. There, the certificates, with a statement regarding the case, signed by a relation of the party desiring the confinement, are sent to the sheriff of the county (the sheriff in Scotland being a judicial officer), who has to satisfy himself, either upon the mere examination of the parties, or, if he thinks proper, by a personal examination of the alleged lunatic, or by calling other evidence, that the alleged lunatic is a proper person to be detained and taken care of. The reasons assigned in favour of this proposition are thus stated by the witness in reply to the question, 'What evils would the course you recommend obviate?' The answer is, 'I think it would give greater security to the public, instead of having an examination after the confinement in an asylum, when the mischief has been done. If you once place a person in an asylum, there is a certain stigma which attaches to him, and which he never gets rid of, and upon persons of weak nerves it has a most prejudicial effect.'

"The two suggestions thus offered to your Committee involve a most important question. But it appears to your Committee, that if either of them were introduced and strictly acted upon, they would be likely to produce still greater evils than those which they profess to remedy. According to the evidence taken before your Committee, it is fully admitted that in a very large majority of cases there is *primâ facie* evidence to justify the confinement. Indeed, it may be said that the instances are extremely rare in which, under the present law, the confinement is or has been unwarranted. If that be so, the evil of acting on the present law without inquiry before a magistrate is more imaginary than real. But the evils arising from a change in that law by insisting on inquiry, when the parties desired it, would often lead to an unnecessary publicity, which it is for the interest of the patient, as well as his family, if possible, to avoid. Insanity under any shape is so fearful a malady, that the desire to withdraw it from the observation of the world is both natural and commendable. The reverse of this would in all instances be painful, and in many it would be cruel. A man in business may become affected with temporary insanity, brought on by over-exertion, mental anxiety, or physical ailment; but if he is early and properly treated, his recovery may be as quick as his seizure was sudden. What could be more injurious than a public inquiry in such cases as these? Where the insanity was undisputed, the inquiry would lead to no useful result, though the knowledge of the malady might be seriously prejudicial to the future prospects of the patient and his family. But when it was disputed, it is unnecessary to dwell on the various mischiefs which would instantly result from it; such, for instance, as the agitations caused to the patient's mind just at the moment when it was trembling on the balance; the injurious comments which might sometimes be made on his character and conduct; the unnecessary exposure of private matters, which need not be brought, and which ought not to be brought before the public gaze, if, at least, it be possible to avoid it; the stigma or prejudice which might permanently attach to him and his children in the event of recovery; and frequently, it may be added, the grievous expense which such inquiries would entail, as they did in the case of Chancery lunatics, where inquisitions were required, until recently, to be held before a jury. Nor should it be forgotten that the delay caused by reference to the magistrate, with a possible inquiry, to be instituted by him into the case, might prevent or retard the immediate treatment which is so requisite for the patient, and thereby tend to aggravate the malady. It ought also to be borne in mind that the sheriff in Scotland is a judicial officer and professionally conversant with legal matters, while a magistrate in England may have little experience in those subjects which, according to this plan, he might be called upon to determine. For these reasons your Committee are disinclined to adopt these suggestions. No doubt the conclusion thus arrived at introduces the further question, what then are the proper safeguards? For if there be even one person improperly confined, it is right to provide the amplest protection which the law can afford in order to prevent so deplorable a result.

"For providing this protection several things are necessary. *In the first place*, it is important that the medical certificate should be clear in its statement, and accurately framed. The whole justification for the patient's confinement depends on this document. The form of the certificate required by law appears to be sufficient; but your Committee are of opinion that some additional security should be taken for ensuring its accuracy. It is sometimes imperfectly filled up, and the patient is then placed under restraint on a document which does not legally justify his detention. Mr. Bolden's suggestion, that these certificates should be verified before a magistrate, so far only as to enable him to determine whether the Act had been complied with, would probably tend to greater caution in this behalf. It would operate as a check on too hasty a conclusion, and obviate the necessity of further examination, without impeding a proper confinement for the purposes of cure, and without entailing that painful publicity which on so many accounts it is desirable to avoid. This suggestion, when thus considered, deserves to be attended to. *In the second place*, your Committee recommend that the certificate authorizing the detention should be limited, in the first instance, to three months, and no more. It is now granted for an indefinite period; but if it were limited to three months in the first instance, 'the effect would be,' as Lord Shaftesbury observes, 'to compel a revision of the case by the family or friends; the relations would be obliged to look again into the matter, as they would know, in all probability, if they did not do so, the patient would be returned upon their hands.' *In the third place*, the order for receiving the patient into the asylum with which the medical certificates are accompanied, should state the time when the person signing it had last seen the patient; and such order should not be effective unless the applicant had himself seen the patient within three months of his signing the order. A case has been brought to the notice of your Committee, where the party applying had not seen the patient for two years, and another where he had not seen him for six times that period. *In the fourth place*, a copy of the order and of the medical certificates upon which the patient is confined should be sent to the Commissioners within twenty-four hours, instead of within seven days, as at present, in order that their attention may be immediately called to any irregularity in these documents; and *in the fifth place*, the patient should, as soon as possible, be visited by the Commissioners, or by some persons acting directly under their authority; so that the patient should have the fullest opportunity of stating his complaints, if he has any to make, and that if he should appear to be improperly confined, immediate means should be taken for his release. A provision of this kind has been sometimes objected to by the proprietors of asylums, upon the ground that it implies suspicion and undue distrust. But the confinement of a person is too serious a matter to allow any feeling of that kind to interfere with the protection which is due to the patient. Moreover; in those asylums which are well-conducted the proprietors have nothing whatever to fear, and asylums which are ill-conducted ought to be controlled. Undoubtedly it is true that, as above shown, the cases in which persons have been improperly confined are extremely rare; but one has happened within the last twelve months. In this case it turned out, when the facts were heard, that the supposed delusion was not a delusion, and the patient was released as soon as visited by the Commissioners. But before that happened, the confinement had lasted for six weeks." (pp. viii.—x.)

3rd. *The Care and Treatment of the Patient while he remains in the Asylum.*—The Committee think that a recommendation of the Commissioners in Lunacy, to the effect that it should be "made compulsory upon the friends of all private patients, whether in mixed, private, and pauper asylums, registered hospitals, or licensed houses, or under separate care as single patients, to visit them, or delegate some one to visit them periodically, and ascertain by personal inspection the accommodation and comforts provided for them," well deserves consideration, "though there may be some practical difficulty in giving full effect to it." (p. x.)

It is suggested also that "the *primâ facie* right both of receiving visits and also of corresponding should be secured to the patients, and should never be refused by the authorities, except on specified grounds; and in that case patients or friends should be at liberty to appeal to the visitors or Commissioners, as the case may be, against such refusal." It is further suggested that power should be given to the Commissioners and visitors "of ordering the temporary discharge, upon trial, of a patient in a private asylum" —a power which the visitors already possess in the case of patients in county asylums. It is added, however, that "a power of this kind, if it be conferred, should of course be exercised with extreme caution." (p. xi.)

L'inconvenience temp. de la visite des parents serait sous la responsabilité directe du medecin traitant.

4th. *The Restoration of the Patient to Liberty as soon as his Case will with safety admit of it.*—The Report states that "it would be an improvement in the law, and an additional protection and security to the patient, if the notice of recovery which is sent to the Commissioners or visitors after a fourteen days' interval, were required to be sent simultaneously with the notice of recovery which is sent to the relations. Such a requirement would secure attention, prevent delay, and enable the Commissioners at once to act in case of neglect." (p. xi.)

The subject of "*Patients in Single Houses*" is next entered upon, and the Committee consider that "it is very desirable that some provision should be made for the superintendence of this class of cases; and there is no better mode of enforcing such a provision than by making it penal for any medical man to receive any such patient without apprising the Commissioners of it. This would place such patients under authority and supervision." (p. xi.)

In respect of *Chancery Lunatics*, the Report, after reciting the advantages derived from the Lunacy Regulation Act of 1853, states that there is one point which is still capable of further improvement. "By the law as it now stands, the Master has the power of summoning a jury if he think that the circumstances require it; but, before he does so, he must go through the whole case. It is clearly advisable that the power of the Master should in this respect be extended; for the preliminary investigation, to a great extent, is useless, and occasions delay and expense." (p. xiii.)

Again, one of the improvements advantageously introduced by the Regulation Act of 1853, was the doing away with the necessity of a special order of reference to the Master in each stage of the case. The Report states that—

"In consequence of this alteration in the law, and the general orders in lunacy issued in pursuance of it, the saving of expenses has been very considerable. In many respects there is reason to believe that this saving may be carried still further; and if the power of making general orders for that purpose is not sufficient, there can be no reason why it should not be enlarged. The cost even of an unopposed application to the Court is about 20*l.*: it would be much less if an original jurisdiction in other cases, besides those which now exist, were given to the Masters. Mr. Barlow recommends, that with regard to leases of the lunatics' property, as well as with regard to the sums allowed for the maintenance of the lunatic, and the mode in which those sums are applied, original jurisdiction might be given to the Master, without the necessity of going to the Court. Mr. Elmer concurs in these recommendations, and he points out other cases in which a similar course might be adopted; such, for instance, as the transfer into Court of money belonging to the lunatic's estate. Mr. Elmer also concurs in the following suggestions, which have been submitted to the Committee by Mr. Enfield, and they appear to the Committee to be worthy of adoption—viz. 1. To assimilate the powers of the Masters in Lunacy to those of the chief clerks in Chancery. 2. To give the Masters the opportunity of oral communication with the superior judges when any explanation is required, or any pending inquiries, in the same way in which explanations take place between the chief clerk and the Vice-Chancellor. 3. To devolve on the Master the duties of seeing that committees of the person are only allowed so much each year as they actually expend in the maintenance of the lunatics, giving the Masters liberty to allow salaries to committees when they see reason; and, 4. To make periodical returns to the Lord Chancellor of the condition of every case under the charge of the Lord Chancellor as regards committees, their accounts, and their sureties." (p. xiii.)

Further, the Committee suggest that the duty of visiting Chancery lunatics should be transferred to the Commissioners in Lunacy, "so that in all cases there should be one supervision and one mode of treatment, with all the appliances which the best and most recent experience could afford, applicable to all the lunatics in the kingdom." (p. xiii.)

The evils which appertain to the present mode of dealing with *Criminal Lunatics*, the Committee conceive will be removed when the State Asylum, now in course of erection at Broadmoor, is opened for the reception of this class of patients. It is recommended, however, that the Commissioners of Lunacy should be required to visit the State Asylum; the Report adding that—

"The extended supervision will be a new guarantee for its good management, while it will assist the Secretary of State to determine in what way the cases shall be dealt with, according as circumstances may justify a partial or total restoration to liberty. On a subject so delicate and difficult as this, a large and continuous discretionary power must be somewhere reposed, and there can hardly be a doubt that the Secretary of State for the Home Department is the proper person in whom such power should be vested. It would not be advisable to limit his power by provisions unduly strict and specific, since this class of cases, more than all others, requires to be dealt with in the most exceptional manner, according to the circumstances which at the time are or may be applicable to each of them." (p. xv.)

The *Composition and Power of the Lunacy Commission* are next considered. The Committee think that, if their recommendations are carried into effect, some alteration in the law as regards the Commission would be required. The Report states that—

"If the visitation of the Chancery lunatics and single patients, and an additional visitation of workhouses and private asylums is required of the Commissioners, increased facilities will be necessary for the discharge of this class of duties. This object might to a certain extent be obtained by enabling a single Commissioner, whether paid or unpaid, to perform the additional duties required, even if it should be thought essential that each of the existing statutory visits should be made by two paid Commissioners. Your Committee cannot but feel some doubt whether it will be in the power of the Board, as at present constituted, efficiently to discharge the increased duties to be entrusted to it. But as they collect from the evidence of the Chairman that the Commissioners themselves are of opinion that they could do so without any permanent addition either to their number or their staff, your Committee have abstained from recommending, without proof of its necessity, that such addition should be made, and also from considering, as in that case it would have been right to do, in what manner any such addition could best be provided." (p. xv.)

It is also recommended "that all the Acts of Parliament relating to this subject should be consolidated in three statutes; one with reference to public asylums, another with reference to private asylums, and another with reference to Chancery lunatics; and that the amendment suggested in the Report should be incorporated in the consolidated statute." (p. 15.)

Finally, the Committee direct particular attention to several minor alterations, which, although not specifically noticed by them, have been suggested in the evidence and papers laid before the Committee by Messrs. Bolden,* Campbell,† Parnell,‡ and Enfield.§

Such, then, is an abstract of the Select Committee's Report, and we propose now briefly to consider the recommendations it embodies, regarding these chiefly from a point of view suggested at the very outset of the Report. "With regard to [more acute cases of lunacy]" the Committee state, "since 50, or 60, or even 70 per cent. are capable of cure, if taken in time, and carefully treated, it is certainly a matter of *primary importance that our legislative provisions should be so framed as to promote the accomplishment of this desirable object.*" (p. iv.) To what extent has this initial proposition influenced the deliberations and conclusions of the Committee?

I.—It is tolerably evident that a knowledge of the status of lunacy in the kingdom would have facilitated the labours of the Committee in coming to any decisions upon the requirements of lunatics. If the number of lunatics still at large among the population, and their civil and social condition, were accurately or approximatively known, it is to be supposed that the great problem of further provision for our insane, and of the legislative interference which might be requisite for promoting or carrying out that provision, could be approached with some prospect of solution. Conversely, in the absence of such information as that suggested, it may be assumed, without much fear of contradiction,

* First Report, Queries 3077—3200.
† Second Report, Appendix No. 1.
‡ Second Report, Appendix No. 3.
§ Second Report, Appendix Nos. 3, 4, 5.

that the most conscientiously devised measures for the relief of lunacy must be empirical.

Now, the figures made use of by the Committee to show the status of lunacy in the kingdom, simply make manifest the accumulation of lunatics in our asylums and the number of pauper lunatics. That is, these figures show solely the amount of lunacy thrown up to the surface of society, but give no information whatever of the amount at large, or of the sources from which the mass of lunatics now subsisting on public charity come. Nay, more, even the statistics laid before and made use of by the Committee, although exhibiting a serious increase of lunatics under charge from year to year, are so imperfectly prepared, that it is impossible to determine from them the relation of the increase to the population at large; that is, whether that increase be real or apparent. The Committee think that the great increase of known lunacy from year to year since 1844, is chiefly due to the fact, that the tendency of legislation and philanthropy since that period has been to bring hitherto neglected cases to light. This may probably be the case, but the question is one which can be, and surely ought to be, for the future, determined by a rightly ordered system of statistics.

Further, the history of our public asylums and their present condition—that is, in so far as they are principally refuges for chronic lunatics—would seem to show that the substratum of neglected cases of lunacy in the kingdom is far from being exhausted; another consideration marking the extreme importance of ascertaining the number and condition of what may be termed our floating population of lunatics.

It will hardly be denied that the deficiencies in our knowledge which we have just indicated, place an almost insuperable bar to our dealing effectively with nascent and early lunacy among the mass of the population, and restrict our curative efforts within very narrow limits. It is, therefore, with the most painful surprise that we find the Committee making use of the highly imperfect statistics laid before them without comment. We had hoped, so patent was the insufficiency of those statistics for all practical, curative, and preventive purposes, that the Committee would have strongly urged upon the Commissioners of Lunacy and the Government the importance of a complete revision, or rather reform, of the statistics of lunacy. We had hoped, also, that the Committee would have seen the propriety of recommending that the status of lunacy should be made a special subject of investigation in the census of 1861; and we believe that a lamentable error has been committed by the omission of such a recommendation, which will be severely felt before the census of 1871. We are aware that many question whether a census-investigation of lunacy in this country would have led to any trustworthy results. We see no just reason to doubt that it would have been less successful in England in 1861 than such an investigation was in Ireland in 1851, and that it is expected to be again in that country in the approaching census. The census of 1861 offered an early and fitting means of determining approximatively the amount of lunacy, and the civil condition of lunatics in the kingdom. It would have given the very elements, the want of which, more than those of any other elements, most impedes our dealing satisfactorily with the practical question of future provision for our lunatics—to wit, the amount and condition of lunatics at large among the population. Yet this question was immediately brought home to the consideration of the Committee (although, sooth to say, passed over unheeded) by the details laid before them, which showed that the present and prospective asylum accommodation among us was barely sufficient to meet the imperative wants of our lunatics, *leaving entirely out of the question the seven thousand and odd lunatics provided for in workhouses.*

The neglect of this question by the Committee will rivet for another period that unfortunate, yet, under present circumstances, almost necessary system which seems to govern the erection of our public asylums—a system which makes them little better than reservoirs for the reception of our surplus lunacy; a system which reduces their curative influence to a minimum; a system which would be paralleled by an attempt to stop the flow of a river, by damming it up where it enters the sea. If we would hope to influence markedly the development of lunacy by curative measures, we shall have to penetrate first towards the more remote sources of the disease among the mass of the people. Those sources are still hidden in darkness; but as we feel our way towards them, we may rest assured that our opportunities for nipping lunacy in the bud will increase in proportion as we advance, while at the same time we shall lay securely under foot the foundations of a sound system of prevention. The first step in such an investigation must be an attempt to ascertain, either by means of a census inquiry or by some other means, the general status of lunacy in the kingdom. This being ascertained, the ground would be clearly mapped out for those further researches, which would be requisite to probe the questions bearing upon the evolution of lunacy among the people to the core.

It is too commonly imagined that an inquiry into the status of lunacy would lead to little else than the results which immediately come from it, and that the practical value of these would not be commensurate with the expense and trouble of the inquiry. This is an error. The ultimate results of the inquiry would be of greater value than the immediate; for they would form the basis for the further and thorough investigation of the conditions fostering lunacy, and, consequently, pave a secure way to the practical dealing with those conditions.

II. Keeping to the text adopted from the Report, it is somewhat puzzling to read that the Committee, "from the evidence" tendered to it, has concluded that our public asylums "are, generally speaking, so well looked after and so carefully attended to, that, as regards them, but little alteration is required in the law." If this exceedingly favourable opinion refers simply to the routine management of the asylums, we could concur in it without hesitation; but if it is meant in any degree to imply that they are to be regarded, in their present state, as models of institutions for the curative treatment of insanity, the conclusion cannot but be regarded as running somewhat counter to the evidence.

It was shown that the tendency of local governing bodies to erect huge buildings, and crowd them with patients, as in the case of Colney Hatch and Hanwell, notwithstanding that all experience had proved that such structures were ill-adapted to the curative treatment of the insane, could not be controlled by the Commissioners in Lunacy, or even the Secretary of State, short of forbidding the construction altogether, if the local governing powers were pleased to carry out their own fancies. It was shown that of 1000 patients in the Hanwell Asylum, not more than 50 could be spoken of as "curable;" and the Chairman of the Committee of Visitors to the Colney Hatch Asylum stated that, "as at Hanwell, the proportion at Colney Hatch supposed to be susceptible of cure is very much the same;" adding, "it must be remembered that we are an asylum rather than an hospital. We are an hospital only in occasional instances, an asylum always." The evidence also went to show that the condition of these two asylums represented, more or less, the condition of almost every public asylum in the country. It was shown, moreover, that the medical and general staff of the majority of public asylums was insufficient for that effective medical and general care of the patients which was essential to their effective curative treatment. In fact, the evidence clearly indicated that, as *hospitals*, our public asylums hold a position lamentably low, compared with the means at their command.

There was no lack of information in the evidence of the causes of this unfortunate state of our great asylums. These might be chiefly traced to erroneous (as we think) notions of economy on the part of the governing bodies, and to the still too prevalent notion among these bodies that an asylum is not necessarily a medical institution. This latter idea is no doubt correct of the original constitution of the majority of our public asylums, but experience has taught us that so long as asylums are regarded primarily as refuges, and only secondarily as hospitals, for the care of the insane, so long the popular aversion to these "lunatic prisons" will be kept up. There can be little doubt that it rests mainly with the governing bodies of public asylums to give to them an entirely medical, and in so far house-of-recovery aspect, and that they have the power to do this. It is certain that *an asylum will never and can never become an hospital for the cure of lunacy, and will never be regarded by the public at large as such, until it becomes a strictly medical institution.*

Few things in the whole course of the official evidence given before the Committee, will exert a more harmful effect than the inconsiderate expressions of opinion tending to depreciate the position of the medical man in relation to lunacy. Such a notion, authoritatively stated, cannot but serve to confirm local governing bodies in their comparative indifference to the curative treatment of the patients in public asylums; and as a consequence will tend in no small degree to perpetuate the idea, which offers so great an impediment to the early reception of patients from among the poor—to wit, that an asylum is a house of detention, not of cure.

Be this as it may, however, the expression of opinion by the Committee, so highly favourable to the present state of our public asylums, and insufficiently guarded by the remarks which follow, can hardly fail to protract a state of things which, according to the evidence before the Committee, was most unfavourable, and in some respects altogether repellant, of the early treatment of cases of lunacy.

It may be that the deficiencies in our public asylums which we have hinted at were not of a nature to be removed by legal enactments. Upon this point we shall offer no opinion; but, after the Committee had asserted the "primary importance" of the early treatment of insanity, it was but just to the House of Commons and the public that the inaptitude of our public asylums to afford that early treatment, and the causes of that inaptitude, should have been fully set forth in the Report.

III. The suggestions of the Committee respecting lunatics detained in workhouses are very important, and will doubtless be carried into effect; but in so far as that detention may interfere with the early treatment of lunatics, it is to be feared that the suggestions would only yield very partial relief, until the deficiencies in our public asylums, already noted, are in a great degree done away with.

The suggestion that the time at which committees of visitors may grant superannuation allowances to their medical officers should be reduced from twenty to fifteen years, is a just acknowledgment of the arduous duties of those men, and we trust will pass into law. We hope also that the strongly-expressed opinions of the Commissioners in Lunacy, examined before the Committee, on the insufficiency of the medical officers' salaries, will not fall to the ground. The important services rendered by the medical superintendents of asylums, often under circumstances of no ordinary difficulty, and the heavy responsibility resting upon them, are as yet but ill-appreciated by the public.

4. The opinion expressed by the Committee on the so-called middle-class asylums, so far as that opinion would imply the desirability of founding asylums which would meet the wants of a class of cases inadequately provided for by existing asylums, is a just one. But the quotation of Lord Shaftesbury's abstract reasoning in favour of the establishment of such asylums, to wit, that they are free from all those evils which the "principle of profit" has introduced into, and which, he conceives, utterly vitiates the management of private asylums, will be apt to lead to the conclusion that the Committee approved of that reasoning. If this were the case, then it would seem that the evidence tendered before the Committee on the working of the chartered asylums of Scotland (the type of middle-class asylum which Lord Shaftesbury would introduce into England), by one of the Scotch Commissioners in Lunacy, must have been to no small extent, if not entirely, unheeded by the Committee.

Dr. Coxe's evidence was sufficient to show that Lord Shaftesbury's statements concerning the value of the system of chartered asylums, and its freedom from the evils which he asserted were inseparably attached to the system of private asylums in England, were, to say the least, exaggerated. The opinions of the Scotch Commissioners in Lunacy as recorded in their last (second) Annual Report, is conclusive on this point. The question is altogether so important, that we shall not hesitate to quote here the opinions referred to, notwithstanding that we have already quoted them in a previous number of the Journal, when reviewing the Report named. The Scotch Lunacy Commissioners state that:—

"During the past year the condition of the public asylums has, on the whole, continued to improve, although, in several respects, it falls considerably below the standard of English county asylums. But, in making this comparison, we must direct attention to the fact, that in one essential respect the Scotch asylums do not occupy nearly so favourable a position as those of England. In the latter country, the necessary funds are raised by assessment; and an asylum, calculated to afford accommodation for all the patients of the county, and supplied with all the necessary appliances, is at once provided. Should this accommodation be afterwards found insufficient, a further assessment is made and additional buildings are erected. In Scotland, on the other hand, the directors of the public asylums possess no compulsory powers of raising funds. The houses have been built with money derived from legacies, charitable donations, and subscriptions; and their extension chiefly provided for by the payments made for patients. The cost of the original building, and its subsequent extension, have thus both been defrayed from uncertain sources; and a considerable portion of the payments for patients has been diverted from the more legitimate object of providing for the proper treatment and comfort of those on whose account they were made, into furnishing accommodation for others. In this way, a large proportion of the public asylum accommodation in Scotland has been provided from moneys levied directly on the friends of the insane, by making the payments on their account considerably exceed the expenditure, instead of by the fairer course of assessing the community. This procedure is well illustrated by the history of the Dundee Asylum. A sum, amounting to 7706*l.* 10*s.* 8*d.*, having been raised by charitable contributions, the asylum was erected at a cost of 8493*l.* 9*s.* 6½*d.* Accordingly, when opened for the reception of patients in 1820, a debt had been contracted of 786*l.* 18*s.* 10½*d.* In 1859 the sum expended on land and buildings had increased to 35,262*l.* 3*s.* 2*d.*, of which sum 5640*l.* 1*s.* 4½*d.* had been obtained through further charitable contributions, and 4144*l.* 8*s.* 9*d.* had been borrowed. It thus appears that during the 39 years which have elapsed since the opening of the asylum, the patients have contributed 17,771*l.* 2*s.* 4½*d.* beyond the cost of their maintenance; and this sum has been spent, not for the special benefit of these patients, but in providing accommodation for the district. In other words, a public want has been supplied from the private funds of those who, perhaps, of all the community were least able to afford the sacrifice." (p. lii.)

Of the celebrated Crichton Institution, at Dumfries, it is said :—

"The plan of the building of the Crichton Institution is such that many of the galleries must necessarily have a dark and gloomy appearance; nevertheless, they are capable of being rendered much more cheerful than they are. There is especially room for improvement of the furniture, in which there is a pervading barrenness, both in quantity and quality, throughout the establishment. It is greatly objected to private asylums that the principle of profit is allowed to interfere with the comforts of the patients; but in the present case, it appears that the necessity of providing funds for the extension of the pauper departments of the asylum is permitted to swallow up an undue share of the payments made for private patients. The apartments of the highest class of patients, though comfortably furnished in essential respects, are scarcely fitted in accordance with the previous habits of the patients, or their position in society; but it is in the lower galleries that the want of articles necessary even for comfort is most apparent, the flagged day-rooms there having neither matting nor carpeting. The lowest rate at which patients are now admitted is 50*l.* a-year." (p. lviii.)

That which is true of the Crichton Institution is also true, to a greater or less extent, of the other chartered asylums of Scotland.

Here then we see (1) that the accommodation provided for the patients in the best Scotch chartered asylums barely equals that provided in the inferior English private asylums, while (2) it is authoritatively stated that a "considerable portion of the payments for patients has been diverted from the more legitimate object of providing for the proper treatment and comfort of those on whose account they were made, into furnishing accommodation for others." Thus it would be easy to show, if we were to adopt Lord Shaftesbury's style of argument, that the "principle of economy" vitiates the whole system of our public asylums, Scotch and English, whether chartered or not; and it would be no difficult task to parade reasons in abundance, seemingly utterly condemnatory of the pecuniary principles governing the working of public asylums, and from the evils arising from the "principle of economy" erect a portentous argument tending to prove that nothing short of the entire abolition of public asylums would destroy those evils. But we are wearied with the reiteration in a grave inquiry of the wretched sophism of the "principle of profit." Each class of asylums (both Scotch and English) has its special advantages, each its peculiar evils. To foster the one and ameliorate or remove the other is the teaching of ordinary prudence; but to attempt to effect this by elevating inordinately, and at the expense of sober fact, the advantages of the one at the expense of the other, must prove as futile in the end, and as injurious in the process, as the efforts of that ingenious individual who sought to lengthen his cloth by adding to the one end that which he had cut off the other.

To the suggestions offered by the Committee for the improvement of the law regulating private asylums, no serious objection will probably be offered. These suggestions do not partake of that vexatious and intermeddling character which too frequently characterized the opinions advanced by several of the witnesses, while at the same time they would, if carried into effect, ensure increased care in the confinement of lunatics, and consequently give greater confidence in this respect to the public. The suggestion that "the patient should as soon as possible be visited by the Commissioners, or by some persons acting under their authority," would alone, perhaps, lead to some difficulty in being carried out, and it is not to be forgotten that this suggestion was originally offered by the Commissioners in Lunacy "only as an expedient, with a view of satisfying public feeling, and not with any hope that it would be really effective."* The Committee

* Report 1, Qy. 81.

rightly state that cases of improper confinement are extremely rare, and it is to be regretted that the solitary case referred to by them was not more fully investigated, so that the possibility of misapprehension might have been put out of the question. We cannot overlook the fact that Mr. Perceval directed the attention of the Committee to a supposed case of unjust confinement, and that on a further examination of the case by the Committee, it was found that that gentleman was labouring under an egregious misconception.* It would have been well to have removed the possibility of doubt from the case recorded by Mr. Bolden.

5. The suggestions respecting patients in single houses, Chancery lunatics, and the Commission, need no comment other than a general approval; those respecting criminal lunatics have already been carried into effect by an Act passed in the session just terminated, and of which an abstract is given on another page.

If now, in concluding these observations, we ask, still keeping chiefly to our text, to what extent the protracted inquiry, conducted by the Select Committee, is likely to promote the earlier and more efficient treatment of lunatics, we are compelled to answer, to little if any. Throughout the whole of the inquiry the chief question has been the protection of the lunatic from unjust restraint, *not from the sad disease from which he suffers.* The lunatic has been persistently regarded as an unfortunate being, constantly subject to unjustifiable control; but only incidentally looked upon as an individual suffering from a curable disorder. If the latter has not been entirely overlooked, it has only been influential so far as to prevent the Committee concurring in suggestions which would add other difficulties to those already existing in the way of the earlier treatment of lunatics. The growing importance of the early and earlier treatment of lunacy was made most clearly manifest in the course of the inquiry, as is very obvious from the text with which we have headed our comments; *yet the question of the possibility of facilitating this early treatment without at the same time weakening the legal protection extended to the unfortunate was never touched upon.* Had it been so, the utter insufficiency of the data before the Committee would at once have become apparent, and the necessity for a complete inquiry into the status and fostering causes of lunacy, previous to further legislation upon the subject, would have been too evident to have been set aside. The hints derivable from the inquiry instituted by the Commissioners in Lunacy, into the condition of lunatics in

* Report 3.

workhouses, should, however, at least have been sufficient to have directed the attention of the Committee to the necessity of more accurate information as to the fostering causes of lunacy among the poorer classes of the population. That inquiry showed a state of things tending to the perpetuation of lunacy among the poor such as had not previously been dreamt of, and which can at once be met by legislation.

We do not for a moment underrate the importance of protecting the lunatic as much as may be practicable from unjust confinement or improper treatment, and so far as the present inquiry will help to secure that result, without casting additional impediments in the way of his medical treatment, we accept the issue as of importance. At the same time we accord no small praise to the cautious manner with which the Committee have dealt with the evidence submitted to them; but we protest against the mode in which, throughout the inquiry, the medical care of the lunatic has been regarded as almost entirely subsidiary to his legal. Had the evidence even shown that the popular belief in the too frequently unjust detention and improper treatment of the insane had been to some extent correct—a belief which, by the way, gave rise to the inquiry, but which the evidence clearly showed to be almost entirely without foundation, in so far as asylums were concerned—this depreciation of the relation of medicine to lunacy would have been an unjustifiable wrong to the lunatic. How much greater then is the injury to the insane when the depreciation is persisted in, after the circumstances which were supposed to justify it, are proved, upon the evidence of those who have chiefly supported and fostered it, to be without any just foundation? If the care and treatment of lunacy are to become *dilettanti* affairs, well and good; but if they are to be looked upon as involving the prevention and cure of lunacy, and that for these purposes a knowledge of lunacy in all its bearings is required, then so long as legislators and philanthropists will deal solely with those questions bearing upon the subject which are obtruded to the surface, so long will inquiries, such as the Parliamentary one just ended, prove abortive in all that more immediately concerns the interest of the nascent or actual lunatic—his Cure.

